# To Subscribers, Readers, and Contributors

**Published:** 1847-12

**Authors:** 


### THE WESTERN JOURNAL.

New Series.—Vol. VIII.—No. VI.

LOUISVILLE, DECEMBER 1, 1847.

### TO SUBSCRIBERS, READERS, AND CONTRIBUTORS.

The present number closes our second series, of eight volumes, and with the opening of the year 1848, we shall commence a new one—the third. The propriety of this succession of series, or *octades*, to coin a word, for which we have the same warrant as the authors of hexade and decade, will be at once admitted, by all who reflect on the condition of society, in the new countries to which we look for subscribers. Daily increasing in population, new physicians, to meet the augmenting demand, are daily entering the profession; and to all such, it must be desirable, in subscribing for the Journal, to begin with a series. That many, therefore, will encourage our publishers, on new year's day, 1848, we hope, and even venture to expect.

To those who have favored us with contributions for the series, now concluding, we cordially return our thanks. Many of their papers have been noticed in other journals, and we trust they will not forget us in future. Those who have not hitherto recorded themselves on our pages, we as respectfully invite to come forward and do it. No well written paper was ever finished without leaving its author a better man, intellectually, than he was at its beginning. The primary and paramount object of a medical journal is the publication of medical and surgical experience; the diversities of which, in our widely extended country, must of course be very great. To present this experience, it is not necessary to write essays or dissertations. Plain historical narrative may embody the whole, and hundreds who might hesitate as to the former, are well qualified for the latter method of writing. We hope that many of them will come to our aid, and while they advance their own fame, elevate the character of our Western profession, and promote the health and happiness of society.

To our brethren on the Lakes and the Gulf of Mexico, we would address a special request. Of the ship fever which has prevailed along the shores of the former, and, of it, and yellow fever, which has infested the coasts of the latter, throughout the past summer and autumn, we desire to obtain full and authentic accounts;—such accounts, as those who have been amidst them, and those only, can give. Shall not this desire be gratified? We see no difficulty in the way, and know of nothing that will be read with deeper interest.

We must, also, speak a word to the physicians and surgeons of the West and South-west, who have served, or are now serving, in the armies which have marched into Mexico. They must not only have seen a vast number of surgical operations, but many of the diseases which affect all armies in hot climates. We trust their experience will not be lost, and that we shall, in due time, be permitted to disseminate much of it abroad.

Arrangements have been made by which we shall continue to receive monthly communications from a correspondent in Paris.

As to our own future efforts, we dare not speak, having a deep consciousness that they have not hitherto been as great as might have been expected of us. Nevertheless, we now feel that we shall enter on our third series with renewed resolution, and that, if sustained and encouraged by our correspondents, we shall be able to render it more worthy of the medical public than the previous series have been.

